# Ouabain Mimics Low Temperature Rescue of F508del-CFTR in Cystic Fibrosis Epithelial Cells

**DOI:** 10.3389/fphar.2012.00176

**Published:** 2012-10-04

**Authors:** Donglei Zhang, Fabiana Ciciriello, Suzana M. Anjos, Annamaria Carissimo, Jie Liao, Graeme W. Carlile, Haouaria Balghi, Renaud Robert, Alberto Luini, John W. Hanrahan, David Y. Thomas

**Affiliations:** ^1^Department of Biochemistry, McGill UniversityMontréal, QC, Canada; ^2^Telethon Institute of Genetics and MedicineNaples, Italy; ^3^Department of Physiology, McGill UniversityMontréal, QC, Canada; ^4^Institute of Protein Biochemistry, National Research CouncilNaples, Italy

**Keywords:** cystic fibrosis, CFTR, trafficking, quabain, microarray, connectivity map, hierarchical clustering, CFBE cells

## Abstract

Most cases of cystic fibrosis (CF) are caused by the deletion of a single phenylalanine residue at position 508 of the cystic fibrosis transmembrane conductance regulator (CFTR). The mutant F508del-CFTR is retained in the endoplasmic reticulum and degraded, but can be induced by low temperature incubation (29°C) to traffic to the plasma membrane where it functions as a chloride channel. Here we show that, cardiac glycosides, at nanomolar concentrations, can partially correct the trafficking of F508del-CFTR in human CF bronchial epithelial cells (CFBE41o-) and in an F508del-CFTR mouse model. Comparison of the transcriptional profiles obtained with polarized CFBE41o-cells after treatment with ouabain and by low temperature has revealed a striking similarity between the two corrector treatments that is not shared with other correctors. In summary, our study shows a novel function of ouabain and its analogs in the regulation of F508del-CFTR trafficking and suggests that compounds that mimic this low temperature correction of trafficking will provide new avenues for the development of therapeutics for CF.

## Introduction

Cystic fibrosis (CF) is caused by mutations in the gene coding for the cystic fibrosis transmembrane conductance regulator (CFTR), which functions as a plasma membrane anion channel (Riordan et al., 1989; Anderson et al., 1991; Kartner et al., 1991). The most common CFTR mutation, F508del (Rommens et al., 1989), causes retention of the mutant in the ER and its premature degradation by the proteasome (Cheng et al., 1990; Jensen et al., 1995). Nevertheless, F508del-CFTR can form functional channels having reduced activity (Dalemans et al., 1991), moreover its trafficking is temperature sensitive and can be partially rescued in many cell types by incubation at low temperature (≤29°C; Denning et al., 1992; Rennolds et al., 2008). It has been estimated that restoring 10–25% of wild-type CFTR (WT-CFTR) activity in patients would alleviate the major symptoms of CF (Pilewski and Frizzell, 1999; Zhang et al., 2009).

Cell-based assays for “correctors” of F508del-CFTR trafficking have identified chemically diverse small molecules that correct trafficking with variable efficiency (Pedemonte et al., 2005; Van Goor et al., 2006; Carlile et al., 2007). Some of these correctors are thought to interact directly with CFTR by acting as stabilizing ligands or “pharmacological chaperones” (Loo et al., 2006; Sampson et al., 2011) or on other known cellular targets, e.g., phosphodiesterases (Dormer et al., 2005; Robert et al., 2008) and histone deacetylases (Hutt et al., 2010). However, for the majority of correctors that have been described, neither the target nor the mechanism of action are known (Lukacs and Verkman, 2012). We have previously reported a novel cell-based HTS assay that measures the appearance of HA-tagged F508del-CFTR at the surface of BHK cells (Carlile et al., 2007). Using this assay in a high throughput screen we identified the cardiac glycoside ouabagenin, an aglycone of ouabain, as a moderately potent corrector of F508del-CFTR trafficking. Cardiac glycosides have long been used to treat congestive heart failure and cardiac arrhythmia, and digoxin is still prescribed for atrial fibrillation and atrial flutter (Prassas and Diamandis, 2008). Cardiac glycosides bind to a highly conserved site on human Na^+^/K^+^-ATPase alpha subunits with a *K*_d_ of ∼18 nM (Wang et al., 2001), which is expected to increase several fold in the presence of physiological potassium concentrations. In cardiac myocytes inhibiting the pump increases intracellular sodium and reduces membrane sodium/calcium exchange, leading to elevation of intracellular calcium and increased contractile force (Hoyer et al., 2011). Moreover, clinical studies also suggest that cardiac glycosides inhibit cancer cell proliferation and have potential as novel therapeutic agents against cancer (Newman et al., 2008).

In addition to its action as an inhibitor of Na^+^/K^+^-ATPase, ouabain has a signaling function at low concentrations (1–10 nM) that is independent of its effect on ion transport (Zhang et al., 2006). Ouabain-bound Na^+^/K^+^-ATPase can trigger slow calcium oscillations and NF-κB activation, thereby preventing cell death and promoting the proliferation and viability of kidney proximal tubule cells (Li et al., 2006). Interestingly, it has been reported that digitoxin and other cardiac glycosides suppress IL-8-dependent lung inflammation in CF lung epithelial cells (Srivastava et al., 2004). The exact mechanisms by which cardiac glycosides modulate cell proliferation, inflammation, migration, and apoptosis are not known (Aperia, 2007; Prassas and Diamandis, 2008).

Here we describe a novel function for ouabain and its analogs which is linked to its signaling functions. Treatment with low concentrations of ouabain resulted in the functional rescue of F508del-CFTR in human CF bronchial epithelial cells, and also in BHK cells and CF mice that are expected to be less sensitive to ouabain inhibition. Moreover the mechanisms of correction by ouabain and its analogs resemble those of low temperature according to transcriptional profiling and analysis of the Connectivity Map (CMAP) for F508del-CFTR trafficking in polarized parental CFBE41o-cells. Significant connectivity was observed between ouabain and low temperature transcriptional profiles obtained in human CF bronchial epithelial cells and this relationship was confirmed by hierarchical clustering analysis of the expression patterns.

These results reveal a new function for ouabain and its analogs as regulators of F508del-CFTR protein trafficking and indicate that cardiac glycosides act by mimicking low temperature rescue. Transcriptional profiling provides insight into corrector mechanisms, and small molecules that mimic the low temperature signature may be useful in developing therapeutics that correct the trafficking defect in CF.

## Results

### Cardiac glycosides correct the trafficking of F508del-CFTR to the cell surface

**Figure 1:**
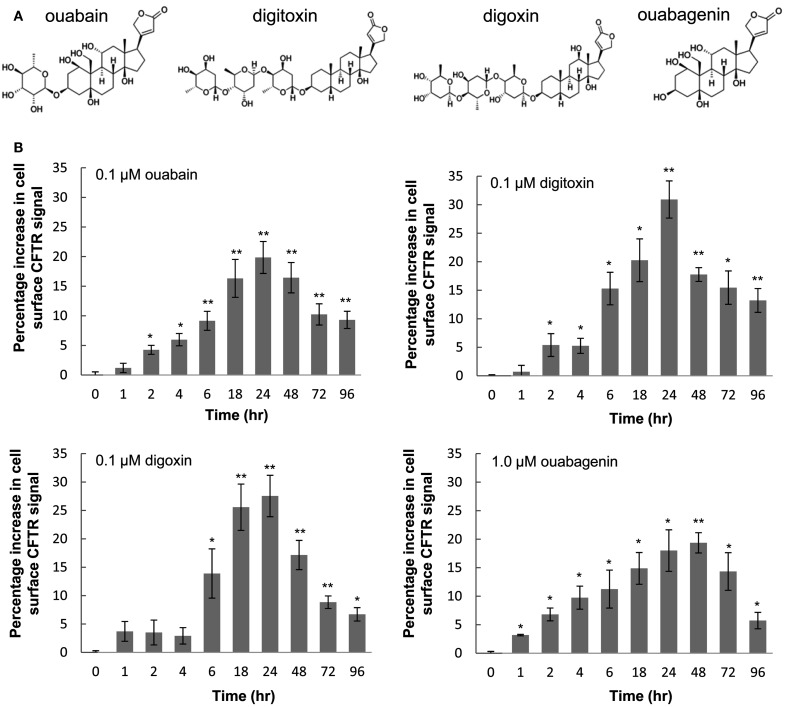
**Cardiac glycosides induce trafficking of HA-tagged F508del-CFTR to the cell surface**. **(A)** Chemical structures of cardiac glycosides tested in this study. **(B)** BHK cells expressing F508del-CFTR-3HA were treated with 0.1 μM ouabain, 0.1 μM digitoxin, 0.1 μM digoxin, and 1.0 μM ouabagenin for various time points or with the vehicle control (0.1% DMSO), the cell surface expression of CFTR were monitored by immunofluorescence assay. Data are presented as percentage increase in surface CFTR signal compared to controls (mean ± SD of *n* = 4; *P < 0.05, **P < 0.005).

From our initial observation that ouabagenin, can correct F508del-CFTR trafficking in BHK cells (Carlile et al., 2007), we selected a panel of structurally related cardiac glycosides, including ouabain, digoxin, and digitoxin (Figure 1A). To confirm that they increase the trafficking of F508del-CFTR to the plasma membrane, we treated BHK cells that express F508del-CFTR-3HA, and measured the appearance of the HA epitope (Carlile et al., 2007). F508del-CFTR-3HA was detectable at the cell surface after 2 h treatment and after 24 h surface expression was increased about 20–30% compared to time 0 h treated cells (Figure 1B).

### Cardiac glycosides improve stability and trafficking of F508del-CFTR

**Figure 2:**
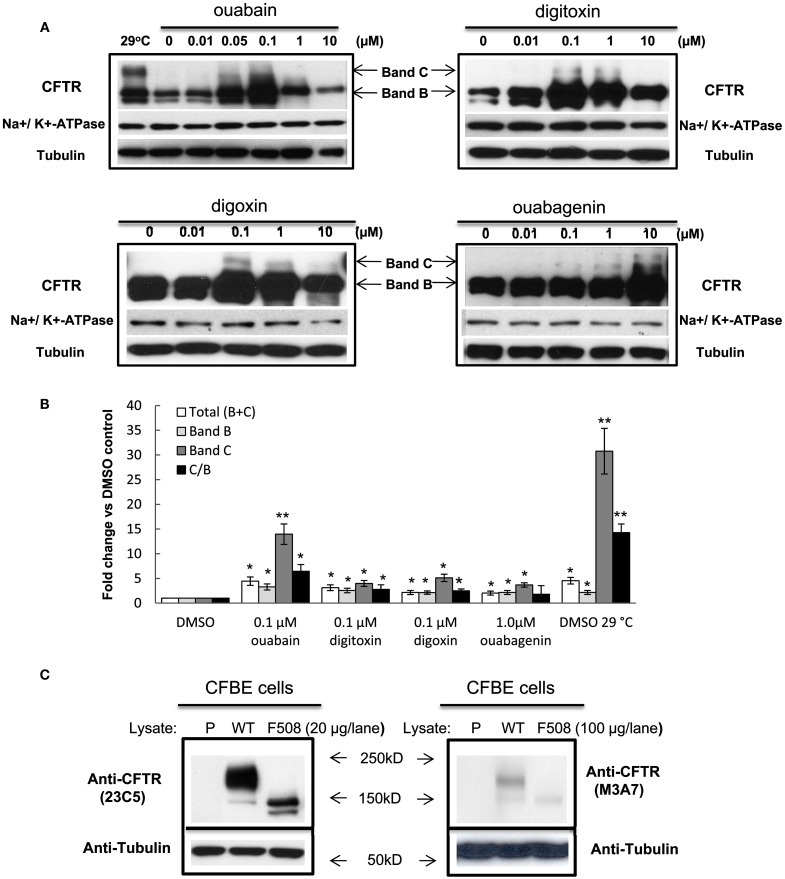
**Cardiac glycosides correct trafficking of F508del-CFTR in CF bronchial epithelial cells**. **(A)** CFBE/F508del-CFTR cells were treated with individual concentrations of ouabain, ouabagenin, digoxin, or digitoxin for 48 h and the cell lysates were analyzed by western blotting using anti-CFTR, anti-Na^+^/K^+^-ATPase alpha1, or anti-tubulin antibodies. CFTR band C and band B are indicated by arrows. Tubulin is shown as a loading control. **(B)** Quantification of the band intensities for **(B)** experiments expressed as fold change vs. DMSO control. Values in the experiments described are presented as means ± SD (*n* = 3). Means were tested for statistical significance using a Student’s *t*-test (*P < 0.05; **P < 0.01). **(C)** The cell lysates from parental CFBE41o- (P), CFBE/WT-CFTR (WT), and CFBE/F508del-CFTR (F508) cells were analyzed using anti-CFTR antibody 23C5 (our own anti-CFTR antibody) or M3A7 (from Chemicon). The molecular weight (kDa, kilodalton) of the markers was shown on the side of the blot.

**Figure 3:**
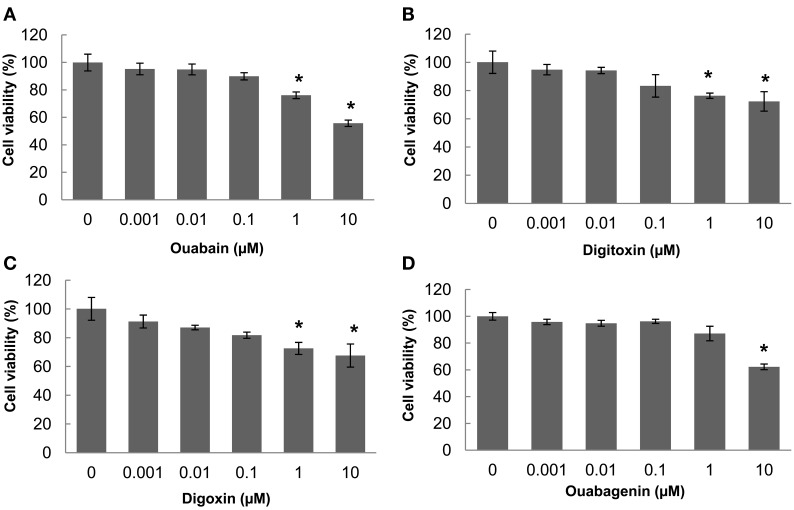
**Nanomolar concentrations of cardiac glycosides have no significant cytotoxic effects on CFBE cells at concentrations that correct CFTR trafficking**. **(A–D)** CFBE/F508del-CFTR cells were treated with different concentrations of each cardiac glycoside for 24 h, the cell proliferations were measured using the AlamarBlue assay. The bar graph shows the percentage of the number of viable cells compared with the number of untreated cells, which were assigned a value of 100% (data shown are the mean ± SD of *n* = 9; *P < 0.05).

The ER-retained glycoform of F508del-CFTR (band B, ∼150 kD) acquires terminal glycosylation (forming band C, ∼175 kD) and traffics through the Golgi. We used immunoblotting to detect the appearance of the mature glycosylated “band C” form of F508del-CFTR in human CF bronchial epithelial cells (CFBE41o-) treated with the individual cardiac glycosides and we evaluated their efficacy in promoting trafficking correction (Figure 2A). We compared the amount of band C with that found in cells treated at low temperature (29°C). Treatment with 100 nM ouabain, digitoxin, or digoxin, or 1 μM ouabagenin, increased the steady-state expression of immature (band B) and mature (band C) glycoforms of F508del-CFTR by 2- to 15-fold above vehicle control (Figure 2B). We also observed an increase in core-glycosylated F508del-CFTR (band B) upon treatment with the cardiac glycosides (Figures 2A,B). There is an overall increase in CFTR protein in the presence of cardiac glycosides, which could result in a “leaky” ER. In order to assess whether the observed increase in trafficking (band C) resulted from ER overload, we calculated the ratio of band C/B (Hutt et al., 2010). We found that all the cardiac glycosides (ouabain, digitoxin, and digoxin) increased the ratio of C/B bands by three- to seven-fold compared with vehicle control (Figure 2B) without affecting Na^+^/K^+^-ATPase protein expression (Figure 2A). In Figure 2C, we compared our own anti-CFTR antibody (23C5) which we have utilized throughout this whole study to the commercial anti-CFTR antibody (M3A7, from Chemicon). The results show that both antibodies gave the similar results, and our own anti-CFTR antibody can detect CFTR bands using much less cell lysates compared with using commercial antibody. To test if these cardiac glycosides have cytotoxicity on CFBE cells or not, in Figure 3, we measured the cell proliferation after 24 h of treatment with each individual cardiac glycoside on CFBE cells, and the results showed that there is no significant cytotoxicity on CFBE cells under 100 nM concentration of cardiac glycosides. Taken together these results show a novel function for ouabain and its analogs in F508del-CFTR folding and trafficking, beyond its well-established role in ion homeostasis.

### Ouabain rescues F508del-CFTR function

**Figure 4:**
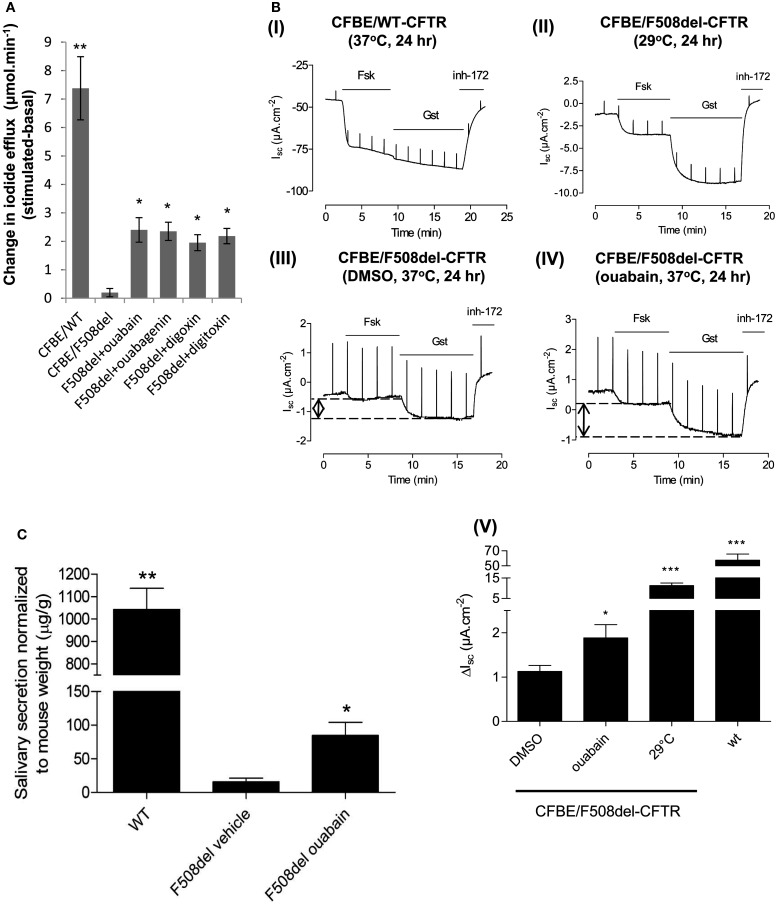
**Ouabain rescues F508del-CFTR channel activity in *in vitro* and *in vivo* assays**. **(A)** CFBE/F508del-CFTR cells treated with ouabain, ouabagenin, digoxin, and digitoxin for 24 h, and iodide efflux was monitored. Data shown are the mean ± SD of *n* = 4 (*P < 0.03; **P < 0.006). **(B)** CFBE/F508del-CFTR cells were treated with or without 0.1 μM ouabain for 24 h and CFTR channel activity was measured by Ussing chamber assay. The Δ*I*_sc_ stimulated by ouabain treatment was compared to low temperature incubation (29°C, 24 h) and to CFBE/WT-CFTR. Histogram showing the change in *I*_sc_ (Δ*I*_sc_) after addition of forskolin + genistein, defined as the difference between the sustained phase of the current response after stimulation and the baseline immediately before stimulation. In the bar graph, data, are presented as mean ± SEM as compared to DMSO control [**(I)** *n* = 9 for CFBE/WT-CFTR cells; **(II)** *n* = 6 for 29°C treatment samples; **(III)** *n* = 8 for DMSO control; **(IV)** *n* = 7 for ouabain; **(V)** *P < 0.05; ***P < 0.001]. **(C)** Salivary secretion in wild-type mice (WT) or F508del-CFTR (F508del) mice treated with vehicle alone or 0.01 mg/kg/day of ouabain for 2 days. Monitored for 30 min following stimulation, results are expressed as the mean ± SEM of *n* = 5 (*P < 0.04; **P < 0.0004).

We next investigated if ouabain and its analogs could also rescue F508del-CFTR channel activity. When CFBE/F508del-CFTR cells were pre-treated with 100 nM ouabain, digoxin, digitoxin, or with 1 μM ouabagenin for 24 h, the iodide efflux response evoked by forskolin increased to levels that were 26–32% that of CFBE cells expressing (WT-CFTR; Figure 4A).

These results were confirmed by measuring the short circuit current (see Materials and Methods) across polarized CFBE/F508del-CFTR cells that had been pre-treated with 100 nM ouabain for 24 h (Figures 4BI–V). A trans-epithelial chloride gradient was imposed and the basolateral membrane was permeabilized using nystatin to ensure that the *I*_sc_ response was mediated by apical Cl^−^ conductance (Robert et al., 2008). Representative *I*_sc_ recordings are shown for WT-CFTR monolayers (Figure 4BI) and F508del-CFTR monolayers pre-incubated for 24 h with normal saline at low temperature (29°C; Figure 4BII), with DMSO vehicle at 37°C (Figure 4BIII), or with 100 nM ouabain at 37°C (Figure 4BIV). Ouabain pre-treatment increased the forskolin and genistein-stimulated *I*_sc_ by ∼1.7-fold compared with controls (Figures 4BIII–V, *P* < 0.05). Chloride current was abolished by the CFTR inhibitor CFTR_inh_-172 (Ma et al., 2002; Caci et al., 2008) in each instance, confirming that the stimulated *I*_sc_ was mediated by CFTR channels. The magnitude of the CFTR-mediated current induced by ouabain (*n* = 7) was 7.5% of that induced by low temperature (*n* = 6), which represents 1.4% of the current measured in cells expressing WT-CFTR (*n* = 9; Figure 4BV).

The correction of F508del-CFTR trafficking and function by ouabain pre-treatment was further evaluated *in vivo* using a CF mouse salivary secretion assay. The F508del-CFTR trafficking defect can be assayed functionally in the ileum and salivary glands of this CF mouse model (French et al., 1996; Robert et al., 2010). Homozygous F508del-CFTR mice and littermate WT controls received continuous low doses of ouabain (0.01 mg/kg/day) or vehicle for 48 h using a micro-osmotic pump implanted under the skin. Salivary secretion was measured acutely by injection of atropine and then isoprenaline into the cheek. Chronic exposure to low levels of ouabain *in vivo* increased the salivary secretion response by ∼5-fold (Figure 4C; *P < 0.04, *n* = 5). This value corresponds to ∼8.1% of the secretory response of littermate WT control mice.

In summary, these data provide evidence that ouabain enhances F508del-CFTR trafficking and channel activity *in vitro* in human CF epithelial cells (CFBE41o-) and *in vivo* in F508del-CFTR homozygous CF mice.

### Ouabain reduces the ER calcium stores in CFBE cells

**Figure 5:**
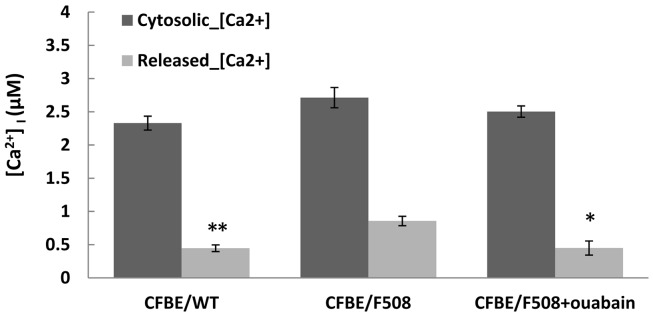
**Ouabain reduced the ER calcium stores in CFBE cells**. CFBE/WT-CFTR and CFBE/F508del-CFTR cells were treated with, or without-, 100 nM ouabain for 24 h. The cells were then loaded with Fura-2/AM, and peak cytosolic Ca^2+^ concentration was measured as the difference in Fura-2 fluorescence recorded before and after adding 2 μM thapsigargin. Data are mean ± SD of *n* = 6 (*P < 0.015; **P < 0.008).

Retention of misfolded proteins in the endoplasmic reticulum is regulated by chaperone proteins, many of which require [Ca^2+^] for optimal activity. Although controversial, several studies have shown that [Ca^2+^] signaling is elevated in CF and that calcium homeostasis in CF airway epithelial cells is disturbed and related to the retention of F508del-CFTR proteins in the ER (Antigny et al., 2008a,b). As the binding of nanomolar concentrations of ouabain to Na^+^/K^+^-ATPase α subunits has previously been reported to increase intracellular calcium (Li et al., 2006; Prassas and Diamandis, 2008), we examined the calcium content of the ER stores in WT-CFTR cells and in ouabain treated vs. untreated CFBE/F508del-CFTR cells (see Materials and Methods). As shown in Figure 5, the cytosolic calcium concentrations in CFBE/WT-CFTR or in CFBE/F508del-CFTR cells are similar before adding thapsigargin. However, after adding thapsigargin, the ER released Ca^2+^ (ER calcium stores) in CFBE/F508del-CFTR cells were about 32% higher than in CFBE/WT-CFTR cells (**P < 0.008, *n* = 6), and ouabain treatment reduced ER calcium stores in CFBE/F508del-CFTR cells by ∼47% (*P < 0.015, *n* = 6). Thus, after 24 h of exposure to a low concentration of ouabain, ER calcium stores in CFBE/F508del-CFTR cells were similar to those in CFBE/WT-CFTR cells. And this normalization of ER [Ca^2+^] in F508del-CFTR cells is also observed by low temperature rescue or by other pharmacological corrections (Antigny et al., 2011).

### Connectivity between ouabain and low temperature transcriptional profiles suggests a similar mode of action

Genome wide transcriptional profiling can be used to infer similarities between the mechanisms of action of different compounds. The CMAP is a rich compendium of 6100 genome wide transcriptional profiles from cultured human cells that have been treated with 1309 bioactive small molecules, including ouabain, and other cardiac glycosides. Gene signatures that show positive correlation with reference profiles (instances) in the CMAP share functional similarities and provide clues to the mechanisms of action of the compounds (Lamb et al., 2006).

**Figure 6:**
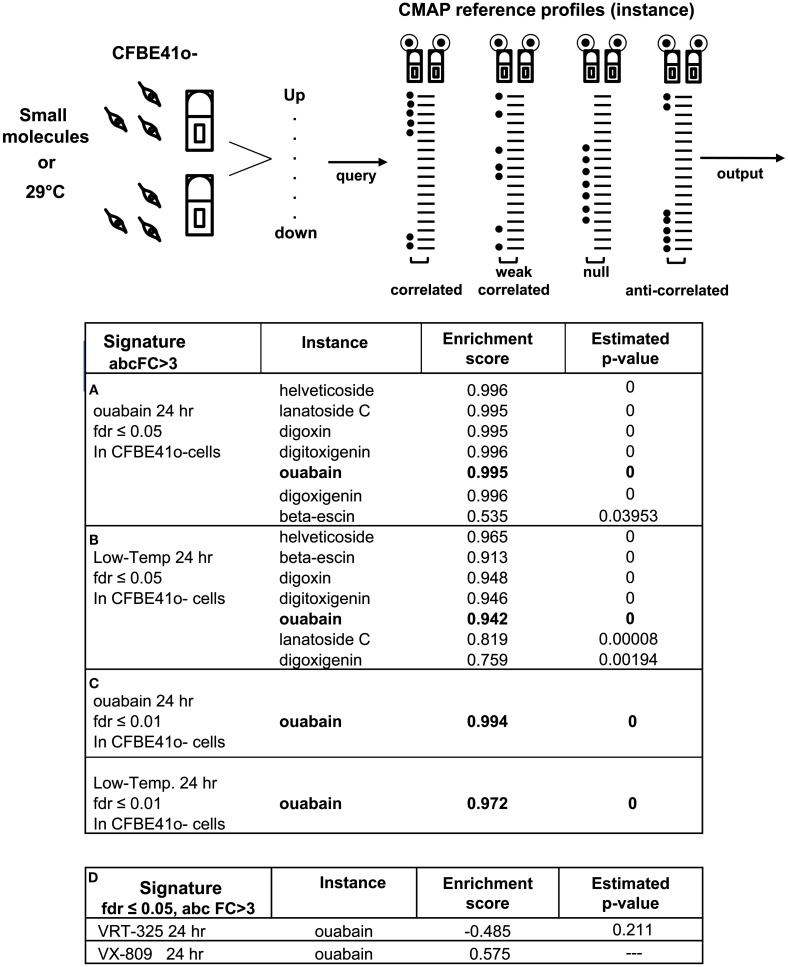
**Ouabain gene expression signature is highly correlated with low temperature**. Cartoon depicting the CMAP concept: pattern-matching algorithms score each established profile for the direction (up- or down-regulated) and strength (fold) of enrichment with the query signature (Lamb et al., 2006). CMAP outputs of 100 nM ouabain (or other small molecules) query signatures obtained in polarized parental CFBE41o-cells treated for 24 h at 37°C and polarized parental CFBE41o-cells treated for 24 h at 29°C (Low-Temp.). Probes generated a FDR ≤ 0.05 and an absolute fold change (absFC) >3 were included in the query signatures. Perturbagens are ordered according to their estimated *P*-values (correlated means = 0) and corresponding connectivity scores, ranging from +1 (correlated) to −1 (anti-correlated). **(A)** The genomic changes induced in polarized parental CFBE41o-cells by ouabain 24 h query signature is correlated with ouabain and six cardiac glycosides previously established profiles in CMAP (instances). **(B)** The genomic changes induced in polarized parental CFBE41o-cells by low temperature 24 h specific-signature is highly ranked with ouabain and six cardiac glycoside instances. **(C)** More stringent query signature (FDR ≤ 0.01) increases the ability of low temperature to recover ouabain instance in the CMAP. **(D)** The genomic changes induced in polarized parental CFBE41o-cells by 10 μM VRT-325 and by 1 μM VX-809 query signatures are respectively weakly anti-correlated and null-correlated with the previously established ouabain profile in CMAP.

To explore the mechanism of F508del-CFTR correction by ouabain, we used transcriptional profiling of parental CFBE41o-cells subjected to different treatments. We generated gene expression profiles using two levels of stringency that were set using a False Discovery Rate (FDR) of ≤0.01 and ≤0.05 (Benjamini and Hochberg, 1995) and an absolute fold Change (absFC) >3. The signatures used to query the CMAP (99 probes up- and 208 probes down-regulated) were from polarized parental CFBE41o-cells treated with ouabain for 24 h. As expected we detected ouabain and six other cardiac glycoside reference profiles (instances) in the CMAP with high confidence (Figure 6A, *P*-value = 0, enrichment score (ES) = 0.995; ES, ranging from +1 means correlated; −1 means anti-correlated). We then queried the CMAP with signatures obtained under three well characterized conditions in which F508del-CFTR trafficking is partially corrected: low temperature rescue (29°C) and the corrector compounds VRT-325 (Loo et al., 2006; Varga et al., 2008) and VX-809 (Van Goor et al., 2011). Remarkably, the low temperature signature (FDR ≤ 0.05; 384 probes up- and 329 probes down-regulated), recovered the ouabain and the other six cardiac glycoside instances with very high ESs (Figure 6B, *P*-value = 0, and ES = 0.942). At higher stringency (FDR ≤ 0.01; 81 probes up- and 74 probes down-regulated), the low temperature signature remained tightly correlated with the cardiac glycoside instances, including ouabain (Figure 6C, *P*-value = 0, and ES = 0.972) suggesting that there is a strong similarity. Conversely, when we queried the CMAP with signatures derived from parental CFBE41o-cells treated with VRT-325 and VX-809, which are thought to act as a pharmacological chaperones that directly bind to F508del-CFTR, we found that the 24-h signature for VRT-325 (FDR ≤ 0.05; 20 probes up- and 139 probes down-regulated) was negatively correlated with ouabain instances and VX-809 24 h signature (FDR ≤ 0.05; 97 probes up- and 64 probes down-regulated) null-correlated with ouabain instances (Figure 6D).

**Figure 7:**
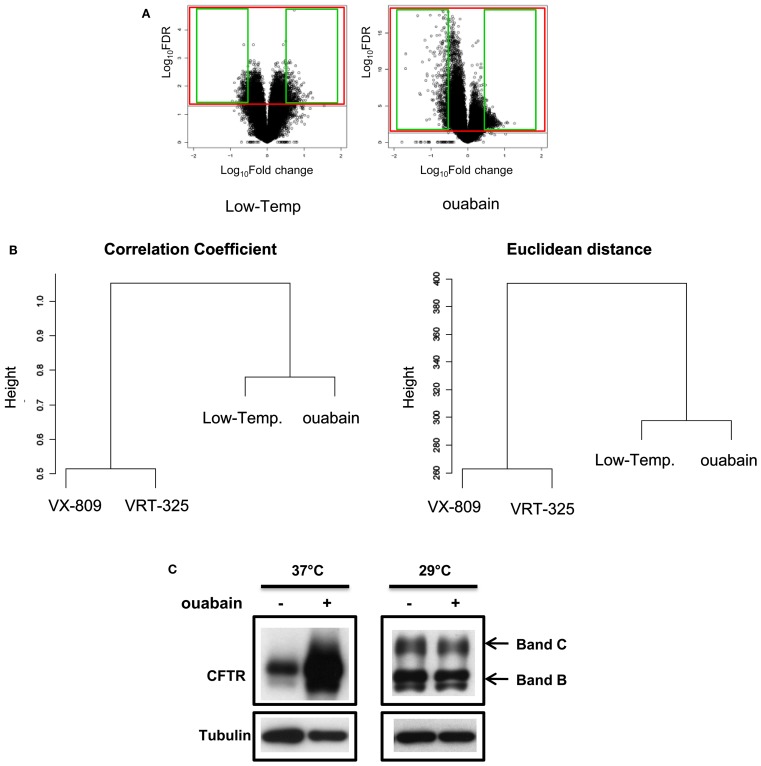
**Ouabain and low temperature rescue have a similar mode of action**. **(A)** Volcano plots of ouabain and low temperature gene expression data. Values are presented as the log_10_ of FDR and fold change. Green squares indicate up- and down-regulated probes for CMAP query signatures for each treatment. Red squares indicate the probes considered for each treatment in the unsupervised hierarchical clustering according to FDR ≤ 0.05. **(B)** Unsupervised hierarchical clustering of ouabain, low temperature, VRT-325 and VX-809 expression patterns (FDR ≤ 0.05) using standard Correlation coefficient and Euclidian distance as similarity measures. The distance between clusters was computed according to complete linkage. **(C)** CFBE/F508del-CFTR cells were treated with or without 0.1 μM ouabain for 48 h at 29 or 37°C, the cell lysates were analyzed by immunoblotting with anti-CFTR or anti-tubulin antibodies.

We next examined a larger number of probes to explore the broader transcriptional response to ouabain and low temperature (31914 probes/41000 Agilent probes, FDR ≤ 0.05; Figure 7A) and to confirm this relationship by measuring the similarity of the expression patterns. We considered the union of the genes that were differentially expressed in each condition and discarded those that were not changed across the four treatments (ouabain, low temperature, VRT-325, and VX-809; FDR ≤ 0.05) in an unsupervised hierarchical clustering analysis (Eisen et al., 1998). In the output from this type of analysis similar patterns of expression are grouped together. Ouabain and low temperature clustered together based on the correlation coefficient and Euclidean distance measurements suggesting they share a similar mechanism of action, whereas VRT-325 and VX-809 form a distinct group, again suggesting that they share a similar mode of action (Figure 7B). To test the hypothesis that ouabain and low temperature rescue operate via a similar mechanism, ouabain treatment of CFBE/F508del-CFTR cells was combined with low temperature incubation in immunoblotting experiments (Figure 7C). No increase in F508del-CFTR trafficking was observed with a combination treatment, suggesting these treatments act in a similar manner and are not additive. Conversely, VRT-325, which stabilized the surface pool of F508del-CFTR as well as corr-4a (Varga et al., 2008), were combined to low temperature treatment and they further improved F508del-CFTR maturation in CFBE/F508del-CFTR cells measured by immunoblotting (Jurkuvenaite et al., 2010; Sondo et al., 2011).

### Ouabain and low temperature treatment generate similar gene expression profiles

**Figure 8:**
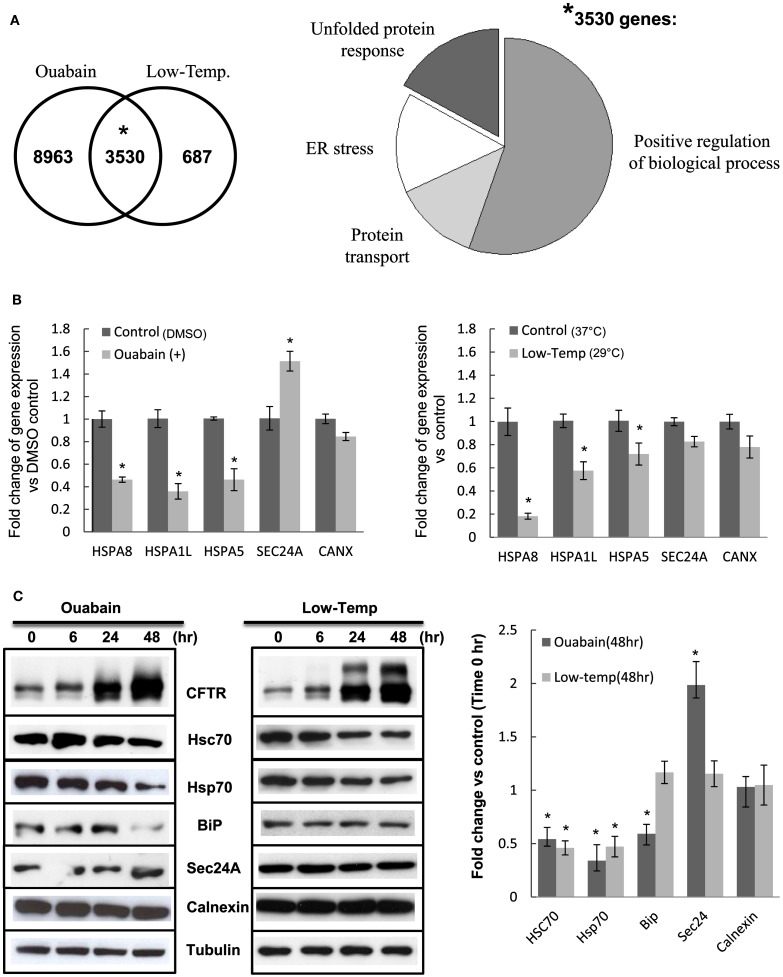
**The effects of ouabain and low temperature on ER-related chaperones**. **(A)** Left panel: Venn diagram of the intersection between ouabain and low temperature 24 h signatures according to a FDR ≤ 0.05. The numbers indicate distinct genes. The probability *P = 0.0001 was calculated using a hyper-geometric random variable. Right panel: Gene Ontology (GO) cellular processes pie chart of 3530 genes in common between ouabain and low temperature 24 h treatments. **(B)** The total RNA previously extracted for the microarray analysis was tested by real-time PCR using the individual primers for the gene expressions of HSPA8, HSPA1L, HSPA5, SEC24A, and CANX. Data are presented by the fold change of gene expression vs. DMSO control with mean ± SEM of *n* = 3 (*P < 0.05) **(C)** CFBE/F508del-CFTR cells were treated with 0.1 μM ouabain or 29°C (Low-Temp.) for 6, 24, or 48 h, then the cell lysates were analyzed by immunoblotting using the individual antibodies. The bar graph shows quantification of the band intensities for blots expressed as fold change vs. Time 0 h control. Values in the experiments described are presented as means ± SD (*n* = 3). Means were tested for statistical significance using a Student’s *t*-test (*P < 0.05).

To gain insight into the molecular processes involved in the stability and trafficking of F508del-CFTR we analyzed the common genes following ouabain and low temperature treatments using the GeneGo Cystic Fibrosis platform (MetaCore^™^ by GeneGo, Inc.). We obtained 3530 genes in common, 8963 unique genes for ouabain and 687 unique genes for low temperature with FDR ≤ 0.05, and the intersection of ouabain and low temperature transcriptional signatures showed that 84% of the differentially expressed genes at 29°C were also differentially expressed with ouabain treatment (Figure 8A, left panel). We performed enrichment analysis to identify functional ontologies in MetaCore with an associated *P*-value (Figure 8A, right panel). Enrichment analysis consists of matching gene IDs of possible targets with those in functional ontologies in GeneGo comparison experiments workflow (Shmelkov et al., 2011).

Remarkably, among the common genes shared between ouabain and low temperature treatments we found that the Gene Ontology (GO) processes that were most highly enriched were: Response to Endoplasmic Reticulum Stress (*P* = 1.11e^−11^), Response to Unfolded Protein (*P* = 1.13e^−11^), Protein Transport (*P* = 1.68e^−11^), and the more general Positive Regulation of Biological Processes (*P* = 1.61e^−9^; Figure 8A, right panel). These results suggest that processes associated with the folding and degradation of CFTR were at the interface between the two treatments. To dissect this further we validated a subset of the 3530 common genes by real-time PCR (Figure 8B), selecting the ones that were most differentially expressed by both treatments and associated with the most enriched GO processes. After treatment with ouabain and low temperature we observed a decrease in the expression of chaperone genes such as *HSPA8*/Hsc70 and *HSPA1L*/Hsp70 that are involved in protein folding and ER-associated degradation (Figure 8B, respectively 60 and 70% reduced by ouabain, 80 and 60% reduced by low temperature; *P-value < 0.05). To test if this altered level of mRNA could also be detected at the protein level we measured the expression of Hsc70 and Hsp70 protein by immunoblotting (Figure 8C). The expression of Hsc70 and Hsp70 chaperones was decreased and correlated with the appearance of mature F508del-CFTR (glycosylated form, band C), and with increased levels of band B (core-glycosylated form; Figure 8C). In contrast, expression of the Unfolded Protein Response (UPR) marker, *HSPA5*/BiP decreased with ouabain treatment but remained unchanged at low temperature, while ER chaperones such as calnexin (tested as a control) were unaltered by ouabain or low temperature (Figures 8B,C). We also observed an increase in the *SEC24A* mRNA and protein expression (Figures 8B,C) following ouabain treatment. Sec24A (COPII complex subunit) implicated in the binding of CFTR destined to traffic from the ER (Routledge et al., 2010) was up-regulated by ouabain treatment but not at low temperature (Figure 8C). At the individual gene level, there were differences in the expression levels between ouabain and low temperature but overall, the striking correlation obtained between the two signatures shows their functional similarities.

## Discussion

Cardiac glycosides have been in clinical use for centuries to treat heart failure, and the mechanism of their positive inotropic effect is well characterized. Ouabain and other cardiac glycosides bind Na^+^/K^+^-ATPase in cardiac myocytes and act by inhibiting its enzymatic activity or down-regulating its expression (Huang et al., 1997; Hoyer et al., 2011). Cardiac glycosides can induce apoptosis and inhibit the growth of cancer cell lines and the pathway to the clinic is expected to be short because the pharmacodynamics and pharmacokinetics of cardiac glycosides are already well-established (Prassas and Diamandis, 2008). Oleandrin, the most promising first generation glycoside-based anticancer drug, is presently in phase I clinical trials to determine the maximum-tolerated dose and evaluate its effect on the pharmacokinetics on chemotherapies administered concurrently (Yang et al., 2009).

In contrast to the apoptotic effects of these drugs on cancer cells, low concentrations of ouabain have also been shown to stimulate the proliferation and inhibit cell death in normal cells (Li et al., 2006). It has been reported that digitoxin and other cardiac glycosides at sub-nanomolar concentrations mimic gene therapy with *CFTR* *in vitro* and can suppress the hypersecretion of IL-8 by cultured CF airway epithelial cells (Srivastava et al., 2004). More recent data show that digoxin derivates attenuate inflammatory lymphocyte function and autoimmune diseases (Huh et al., 2011).

Na^+^/K^+^-ATPase, the target of cardiac glycosides can, in the presence of nanomolar concentrations of ouabain, act as a signal transducer. For instance, it has been reported that ouabain-bound Na^+^/K^+^-ATPase can, independent of its ion transport function, induce multiple signaling pathways including c-Src and intracellular calcium oscillations (Zhang et al., 2006). Several studies have shown that [Ca^2+^] is elevated in CF and becomes normalized when the trafficking of F508del-CFTR is corrected by small molecules or low temperature (Norez et al., 2006, 2009; Antigny et al., 2008a). The relationships between CFTR and calcium signaling have recently been reviewed (Antigny et al., 2011), however the role of [Ca^2+^] in protein biogenesis and trafficking remains incompletely understood. We confirmed the elevation of ER calcium stores in CF cells and showed that ouabain reduces store calcium to normal levels in CFBE cells expressing F508del-CFTR (Figure 5).

Here, we demonstrate that nanomolar ouabain increases F508del-CFTR trafficking to the cell surface and partially restores its function in a human CF bronchial epithelial cell line. Ouabain has this effect without causing substantial inhibition of Na^+^/K^+^-ATPase protein expression or cell viability. Moreover, our *in vivo* results also showed functional rescue of mutant CFTR by ouabain in CF mice and the value corresponds to ∼8.1% of the secretory response of littermate WT control mice and without affecting the mice body weight (control group: starting, 26.36 ± 2.49 g; after 48 h, 26.53 ± 2.63 g, *P* > 0.2; ouabain treated group: starting, 26.98 ± 2.39 g; after 48 h 27.29 ± 2.33 g, *P* > 0.2). Thus our data raise the possibility that cardiac glycosides not only increase total CFTR protein expression, but may also increase the folding yield and trafficking of F508del-CFTR. Ouabain thus joins a growing list of F508del-CFTR corrector compounds that act by modulating proteostasis (Calamini et al., 2012) rather than by acting as pharmacological chaperones that bind to F508del-CFTR (Sampson et al., 2011). Considering the inhibition function of cardiac glycosides on inflammation in cultured CF airway epithelial cells (Srivastava et al., 2004), also associating the long history of cardiac glycosides have being used in clinical treatment, it suggests that ouabain and other cardiac glycosides may have potential therapeutic perspectives for CF patients.

Transcriptional profiling analysis and the CMAP uncovered interesting similarities between very different corrector mechanisms (Lamb et al., 2006). We proved the ability and reliability of the CMAP to connect our ouabain signature and the ouabain reference profiles already present in the CMAP collection, and then found that the resulting transcriptional profile obtained by ouabain treatment resembled that produced by low temperature (29°C) suggesting a similar mechanism of action. The CMAP is a resource that can be used to discover functional connections with a limited number of probes that are up- or down-regulated (≤1000 probes). To better identify the state of the CFBE41o-cells throughout their responses to ouabain, low temperature, VRT-325 and VX-809, we applied an unsupervised hierarchical cluster analysis to obtain a direct measure of similarity of parental CFBE41o-expression patterns. In this approach, a larger number of probes, comparing the ones queried in CMAP, were computed using standard Correlation coefficient and Euclidian distance as measures of similarity. The output groups together genes with similar patterns of expression by a direct measure of similarity and probes which encode for genes that are co-expressed share common mechanisms. We integrated VRT-325 and VX-809 profiles in the clustering analysis not only because both these compounds are thought to bind to F508del-CFTR (Loo et al., 2006; Varga et al., 2008; Van Goor et al., 2011) but also to delineate different “categories” of correctors based on shared mechanisms of action. The addition of this condition enhances our observation by which ouabain and low temperature share a common mechanism and VRT-325 cluster together with VX-809 and we supported our mode of action predictions based on gene expression profiles by combination treatments.

Our study indicates that ouabain and low temperature rescue of F508-CFTR may involve the down-regulation of chaperones (*HSPA8*/Hsc70 and *HSPA1L*/Hsp70), thereby reducing F508del-CFTR degradation; and the up-regulation of COPII components for vesicular export to the Golgi. These two heat shock proteins play important roles in the biosynthesis and degradation of CFTR and it has been shown that a decrease in expression of Hsc70 (in association with the co-chaperone CHIP) results in decreased degradation of F508del-CFTR (Rab et al., 2007; Matsumura et al., 2011). Moreover, we found an increased expression of *SAR1A* (COPII complex subunits; see Supplementary Material) by low temperature incubation. *SAR1A*, together with *SEC24A*, which is up-regulated by ouabain, led to anterograde export of the binding protein to the Golgi (Yoo et al., 2002; Wang et al., 2008). The intersection of ouabain and low temperature transcriptional responses indicates that 84% of the genes that are differentially expressed at 29°C are also differentially expressed with ouabain (Figure 8A, left panel). Thus, ouabain may shift the cells to a “permissive” state by mimicking low temperature, thus correcting the F508del-CFTR folding and trafficking defect.

Dissection of the molecular events that underlie rescue by low temperature and mimicking it with a small molecule may be a strategy to identify CF therapeutics. Galietta and colleagues have shown using CFBE41o-cells that low temperature can synergize with correctors such as corr-4a and VRT-325 (Sondo et al., 2011). Therefore our finding suggests that combination treatments that include ouabain may also be synergistic in the treatment of CF.

In summary, our study shows that low concentrations of ouabain can rescue F508del-CFTR by mimicking low temperature rescue in human CF bronchial epithelial cells. Apart from pharmacological chaperones that bind directly to F508del-CFTR the target of and the mechanism of action of most correctors is unknown. Certainly cardiac glycosides are being investigated for use as a cancer therapeutic and for other diseases (Prassas and Diamandis, 2008). We predict that the Na^+^/K^+^-ATPase or its downstream pathway will be a good place to search for F508del-CFTR correctors.

## Materials and Methods

### Cell culture and transfections

The parental CFBE41o-cell line was originally developed by immortalization of CF (F508del/F508del) bronchial epithelial cells by Dr. D. Gruenert (Kunzelmann et al., 1993). The mutated protein is expressed at low levels in this cell line, therefore two derivatives were generated by transduction using the TranzVector lentivirus system (Wu et al., 2000) to create CFBE/WT-CFTR and CFBE/F508del-CFTR cell lines in which the CFTR protein can be detected by immunoblots. Those cells were generously provided by Dr. J. P. Clancy (University of Alabama, Birmingham) and cultured in EMEM medium supplemented with 10% FBS. Polarized CFBE41o-cells were cultured initially under liquid–liquid conditions, then allowed to polarize at the air–liquid interface. BHK cells stably expressing F508del-CFTR-3HA were cultured as described previously (Carlile et al., 2007).

### The cell-based trafficking assay

The surface expression of CFTR was measured as described previously (Carlile et al., 2007). BHK cells stably expressing F508del-CFTR-3HA (bearing a 3HA-epitope tag in the fourth extracellular loop) were treated with cardiac glycosides. Cells were fixed with 4% paraformaldehyde for 15–20 min at 4°C and incubated with monoclonal anti-HA antibody (Sigma, Canada) solution containing 1% FBS at 4°C overnight. After washing, the plates were analyzed using a plate reader (Analyst^™^ HT 96.384, Biosystems, USA; 488 nm excitation, 510 nm emission) to measure background fluorescence, then incubated with anti-mouse IgG antibody conjugated with FITC (Sigma, Canada) at a dilution of 1:100 for 1 h. The cells were washed, then incubated with 100 μl of PBS, and reanalyzed. The mean fluorescence of 12 mock (DMSO) treated wells was used as the background signal and designated 0% cell surface signal. The surface CFTR signal of cells expressing WT-CFTR on the same plate was designated 100%. The compound treated cell fluorescent signal was then given a percentage value relative to these two controls. Control experiments indicated that the vehicle did not affect trafficking when added alone (data not shown).

### Immunoblotting and antibodies

Cells were lysed in RIPA buffer containing 1% Triton X-100, 0.1% SDS, 150 mM NaCl, 20 mM Tris-HCl (pH 8.0), and 0.08% deoxycholic acid, and lysates were separated by 6% SDS-PAGE and transferred to nitrocellulose filters. The filters were probed with monoclonal anti-tubulin (Sigma), anti-CFTR (monoclonal antibody 23C5, P. Määttänen, M. Mirza, and D. Y. Thomas, unpublished results), anti-CFTR (M3A7, Chemicon), anti-BiP (BD Transduction Laboratories), anti-Hsp70 (Stressgen), rabbit polyclonal anti-calnexin (kindly provided by Dr J. J. Bergeron, McGill University), rabbit anti-Sec24A (Novus Biologicals), and rabbit anti-Hsc70 antibodies (StressMarq). Horseradish peroxidase (HRP)-conjugated secondary antibodies were used and blots were developed using the ECL detection system (Roche, Germany) and exposed to film (Amersham). The films were scanned and analyzed by densitometry using Photoshop (Adobe, Inc.). Quantification of the band intensities for Figures 2B and 8C experiments expressed as fold change vs. DMSO control and normalized by tubulin bands.

### Cytotoxicity assay

The cytotoxic effects of ouabain or other cardiac glycosides (from Sigma) were determined by using the colorimetric AlamarBlue^™^ (Biosource, Camarillo, CA, USA) assay, according to the manufacturer’s instructions. Briefly, cells were plated in triplicate at a density of 3 × 10^5^ cells/per well in 96-well plates and cultured overnight. Cells were then treated with the different concentrations of ouabain or other cardiac glycosides for 24 h. The medium was removed after 24 h and cells were incubated in fresh medium at 37°C, 5% CO_2_ for 4 h. At the end of the 4-h incubation, 10 μl of AlamarBlue was added to each well and incubated at 37°C, 5% CO_2_ for 18 h. Absorbance was measured at 570 and 600 nm and medium without cells was used as blank. Percent survival was quantified according to the manufacturer’s instructions and the untreated sample was set to 100%. Final percent survival was averaged from three triplicates from three independent experiments.

### Statistics

Values in the experiments described are presented as means ± SD. Means were tested for statistical significance using the Student’s *t*-test.

### Iodide efflux assays

Cystic fibrosis transmembrane conductance regulator channel activity was assayed by measuring iodide efflux with a robotic liquid handing system (BioRobot 8000, Qiagen, USA) using Qiagen 4.1 Software as described previously (Robert et al., 2008). Cells were seeded in 24-well plates allowed to reach 100% confluence, and treated with drug or vehicle for an additional 24 h. Cells were then incubated in iodide loading buffer [136 mM NaI, 3 mM KNO_3_, 2 mM Ca(NO_3_)_2_, 11 mM glucose, and 20 mM Hepes pH 7.4] for 1 h at 37°C, then washed with efflux buffer [136 mM NaNO_3_, 3 mM KNO_3_, 2 mM Ca(NO_3_)_2_, 11 mM glucose, and 20 mM Hepes, pH 7.4] and the appearance of *I*^−^ was measured after replacing the buffer at 1 min intervals before and during stimulation with 50 μM genistein and 10 μM forskolin using an iodide-sensitive electrode (Orion Research, Inc., Boston, MA, USA). Relative iodide efflux rates were calculated from the difference between the maximal (peak) iodide concentration during stimulation and the minimal iodide concentration before stimulation.

### Ussing chamber studies

Cystic fibrosis transmembrane conductance regulator channel activity was measured in Ussing chambers as described previously (Robert et al., 2010). Briefly, 2 × 10^6^ cells (CFBE/WT-CFTR or CFBE/F508del-CFTR cells) were seeded onto fibronectin-coated Snapwell 12-mm inserts (Corning Incorporated, Life Sciences, NY, USA) and the apical medium was removed the following day to create an air–liquid interface. Trans-epithelial resistance was monitored using an EVOM epithelial volt ohm meter (World Precision Instruments, Sarasota, FL, USA) and cells were used when the trans-epithelial resistance of the monolayer was 300–400 Ω cm^2^. In some experiments, CFBE/F508del-CFTR monolayers were grown at 29°C or treated with a test compound at 37°C for 24 h before being mounted in chambers and voltage-clamped using a VCCMC multichannel current-voltage clamp (Physiologic Instruments, San Diego, CA, USA). Apical membrane conductance was functionally isolated by permeabilizing the basolateral membrane with 200 μg/ml nystatin and imposing an apical-to-basolateral Cl^−^ gradient. The apical bathing solution contained 115 mM NaCl, 25 mM NaHCO_3_, 1.2 mM MgCl_2_, 1.2 mM CaCl_2_, 2.4 mM KH_2_PO_4_, 1.24 mM K_2_HPO_4_, 10 mM mannitol (pH 7.4 with NaOH). The basolateral bathing solution contained 1.2 mM NaCl, 115 mM Na-gluconate, 25 mM NaHCO_3_, 1.2 mM MgCl_2_, 4 mM CaCl_2_, 2.4 mM, KH_2_PO_4_, 1.24 mM K_2_HPO_4_, 10 mM glucose (pH 7.4 with NaOH). CaCl_2_ was increased to 4 mM to compensate for its chelation by gluconate. The apical solution contained mannitol instead of glucose to eliminate current mediated by Na^+^-glucose cotransporters. Successful permeabilization of the basolateral membrane under these conditions was obvious from the reversal of *I*_sc_. Solutions were continuously gassed and stirred with 95% O_2_-5% CO_2_ and maintained at 37°C. Ag/AgCl reference electrodes were used to measure trans-epithelial voltage and pass current. Pulses (1 mV amplitude, 1 s duration) were imposed every 90 s to monitor resistance. The voltage clamps were connected to a PowerLab/8SP interface (ADInstruments, Colorado Springs, CO, USA) for data collection. Ten micromolars forskolin +50 μM genistein were added to the apical bathing solution to activate CFTR.

### Salivary secretion

The salivary secretion assay was performed as described (Best and Quinton, 2005). Briefly, homozygous Δ508-CFTR mice (*Cftr^tm1^ *Eur**) and WT mice were 10–12 weeks old and when used weighed 20–25 g. A micro pump (Alzet Model 1003D) was fixed under the skin on the back of mouse to deliver a very low dose of ouabain (0.01 mg/kg/day) or vehicle for 48 h. Mice were anesthetized using ketamine and diazepam and 1 mM atropine was injected subcutaneously into the left cheek to block cholinergic responses. After absorbing any saliva with Whatman filter paper, 100 μM isoprenaline was injected at the same site with 1 mM atropine to induce secretion and saliva was collected on filter paper every 3 min for 30 min. Samples were immediately sealed in a pre-weighed vial and the saliva secretion rate and the total amount were normalized to mouse weight. All procedures were performed according to guidelines developed by the Canadian Council on Animal Care and the protocol was approved by the McGill University Animal Care Committee.

### ER calcium store measurements

Thapsigargin-releasable ER calcium was calculated as the difference in cytoplasmic calcium measured before and after the addition of 2 μM thapsigargin to cells in Ca^2+^-free buffer. In brief, the cells were grown and treated with or without 0.1 μM ouabain for 24 h, then 2 × 10^6^ cells (CFBE/WT-CFTR or CFBE/F508del-CFTR cells) were harvested and washed in Ca^2+^-free buffer (20 mM HEPES, pH 7.4, 143 mM NaCl, 6 mM KCl, 1 mM MgSO_4_, 0.1% glucose, 0.1% bovine serum albumin, 250 mM sulfinpyrazone). The cells were resuspended in 200 μl of calcium-free buffer containing 0.02% pluronic acid and subsequently loaded with the cell-permeable fluorescent indicator Fura-2/AM at 3 mM for 30 min at 37°C. After a final wash, the cells were resuspended in Ca^2+^-free buffer and a 340/380-nm excitation ratio at a 510-nm emission wavelength were obtained using a LS 50B PerkinElmer Life Sciences luminescence spectrophotometer. The fluorescence ratio (340/380) was measured in cells treated with 2 μM thapsigargin and the Fura-2 ratio values converted to [Ca^2+^] according Grynkiewicz et al. (1985). The peak of thapsigargin-releasable [Ca^2+^]_cyto_ was calculated as the difference in cytoplasmic calcium measured before and after the addition of 2 μM thapsigargin to cells in Ca^2+^-free Hanks’ buffer.

### Microarray analysis

Polarized parental CFBE41o-cells cultured at the air–liquid interface were used for microarray assays. RNA samples were extracted in 1 ml TRIzol Reagent (Invitrogen, USA), quantificated by spectrophotometry (Nanodrop, USA), and RNA integrity was assessed using an Agilent 2100 Bioanalyzer (Agilent Technologies, Santa Clara, CA, USA). Only samples with an RNA integrity number (RIN) ≥ of 8 were used for amplification. Total RNA (1 mg) was subjected to two rounds of amplification using the Amino Allyl MessageAMP II aRNA amplification kit (Ambion, Applied Biosystems, USA). The integrity and quantity of the aRNA was revaluated by Nanodrop and Agilent Bioanalyzer, and coupled to Cy3 and Cy5 (Amersham Biosciences, UK). Whole Human Genome 44 K arrays (Agilent Technologies, product G4112A) were used for all experiments. RNA samples (825 ng/each) were subjected to fragmentation followed by 16 h hybridization, washing, and scanning (Agilent Technologies, model G2505B) according to the manufacturer’s protocol (manual ID #G4140-90030). Samples were hybridized against Universal Human Reference RNA (Stratagene, ID #740000, La Jolla, CA, USA). Duplicate hybridizations were performed for each sample using reverse-dye labeling. Arrays were washed according to manufacturer’s recommendations, scanned using an Agilent dual-laser microarray scanner (Model G2505B), and Cy5/Cy3-signals were quantified using Agilent’s Feature Extraction software (v.7.11) with the default parameters.

Microarray quality control reports generated by the Agilent Feature Extraction software were used to detect hybridization artifacts. Probe level raw intensities were processed using R/BioConductor and Limma package (Gentleman et al., 2004). Data were background corrected using “normexp” limma method and normalized in two steps: loess normalization within-array to correct systematic dye-bias and quantile normalization between–arrays to detect systematic non-biological bias. Ratios representing the relative target mRNA intensities compared to Universal Human Reference RNA probe signals were derived from normalized data. To remove “batch effects” across microarray experiments we adjusted the data using the empirical Bayes method available at: http://biosun1.harvard.edu/complab/batch/ (Johnson et al., 2007). PCA plots and Clustering trees of normalized adjusted intensities were drawn for each time-point specific sets of samples to confirm the robustness of the method used.

To find differentially expressed genes (treatment vs. control), a *t*-test was applied for each time-point. For each *P*-value, the Benjamini–Hochberg procedure was used to calculate the FDR (Benjamini and Hochberg, 1995). Genes were considered to be differentially expressed if the corrected FDR ≤ 0.05 (while controlling the expected FDR to no more than 5%). Unsupervised hierarchical clustering was performed on normalized data (FDR ≤ 0.05), with complete linkage and Euclidian and Pearson’s correlation distances.

### Functional category enrichment analyses

Identification of overrepresented functional categories (pathways and cellular processes) was performed per treatments using the complete set of differentially expressed genes (FDR ≤ 0.05) in the MetaCore^™^ suit (Version 6.1; GeneGo, Inc., St. Joseph, MI, USA; Nikolsky et al., 2005). The functional analysis were based on MetaCore’s proprietary manually curated data base of CF specific contents (Nikolsky et al., 2009).

### Real-time PCR

Total RNA was extracted from cells using TRIzol Reagent (Life Technologies, Inc., Burlington, ON, Canada), and the cDNA was synthesized using AffinityScript QPCR cDNA Synthesis Kit (Stratagene, La Jolla, CA, USA). Real-time PCR was performed using a Stratagene Mx3005PTM system (Stratagene, La Jolla, CA, USA) as follows: 20 μl reaction solution contained 10 μl SYBR Green Supermix (Bio-Rad Laboratories, Inc., Hercules, CA, USA); 0.4 μl sense and reverse primer (25 ng/μl); 2 μl diluted cDNA; 7.2 μl nuclease-free water. For the cross-validation real-time PCR experiments we used the same total RNA extracted for the microarray assays. The primer sequences were designed according to the GenBank^™^ accession numbers: GAPDH NM_002046; CFTR NM_000492; HSPA8 NM_006597; HSPA1L NM_005527; HSPA5 NM_005347; SEC24A NM_021982; and CANX NM_001746. The subsequent data analysis was performed using MxPro^™^ QPCR Software followed by comparative quantification real-time PCR. Gene expression levels were normalized to GAPDH gene expression and compared with untreated control, which was assigned a value of 1.

## Conflict of Interest Statement

The authors declare that the research was conducted in the absence of any commercial or financial relationships that could be construed as a potential conflict of interest.

## Supplementary Material

The Supplementary Material for this article can be found online at http://www.frontiersin.org/Pharmacology_of_Ion_Channels_and_Channelopathies/10.3389/fphar.2012.00176/abstract

## Abbreviations

CF, cystic fibrosis; CFBE, cystic fibrosis bronchial epithelial; CFTR, cystic fibrosis transmembrane conductance regulator; CMAP, connectivity MAP; COPII, vesicle coat proteins; ER, endoplasmic reticulum; ES, enrichment score; FDR, false discovery rate; HA, hemagglutinin; HTS, high throughput screening; Na^+^/K^+^-ATPase, sodium-potassium adenosine triphosphatase; NF-κB, nuclear factor kappa-light-chain-enhancer of activated B cells.

## References

[B1] Anderson M. P. Berger H. A. Rich D. P. Gregory R. J. Smith A. E. Welsh M. J. (1991). Nucleoside triphosphates are required to open the CFTR chloride channel. Cell 67, 775–784 10.1016/0092-8674(91)90072-7 1718606

[B2] Antigny F. Norez C. Becq F. Vandebrouck C. (2008a). Calcium homeostasis is abnormal in cystic fibrosis airway epithelial cells but is normalized after rescue of F508del-CFTR. Cell Calcium 43, 175–183 10.1016/j.ceca.2007.05.002 17590432

[B3] Antigny F. Norez C. Cantereau A. Becq F. Vandebrouck C. (2008b). Abnormal spatial diffusion of Ca2+ in F508del-CFTR airway epithelial cells. Respir. Res. 9, 70 10.1186/1465-9921-9-70 18973672 PMC2584091

[B4] Antigny F. Norez C. Becq F. Vandebrouck C. (2011). CFTR and Ca signaling in cystic fibrosis. Front. Pharmacol. 2:67 10.3389/fphar.2011.00067 22046162 PMC3200540

[B5] Aperia A. (2007). New roles for an old enzyme: Na,K-ATPase emerges as an interesting drug target. J. Intern. Med. 261, 44–52 10.1111/j.1365-2796.2006.01756.x 17222167

[B6] Benjamini Y. Hochberg Y. (1995). Controlling the false discovery rate – a practical and powerful approach to multiple testing. J. R. Stat. Soc. Ser. B Stat. Methodol. 57, 289–300

[B7] Best J. A. Quinton P. M. (2005). Salivary secretion assay for drug efficacy for cystic fibrosis in mice. Exp. Physiol. 90, 189–193 10.1113/expphysiol.2004.028720 15572461

[B8] Caci E. Caputo A. Hinzpeter A. Arous N. Fanen P. Sonawane N. (2008). Evidence for direct CFTR inhibition by CFTR(inh)-172 based on Arg347 mutagenesis. Biochem. J. 413, 135–142 10.1042/BJ20080029 18366345

[B9] Calamini B. Silva M. C. Madoux F. Hutt D. M. Khanna S. Chalfant M. A. (2012). Small-molecule proteostasis regulators for protein conformational diseases. Nat. Chem. Biol. 8, 185–196 10.1038/nphys2194 22198733 PMC3262058

[B10] Carlile G. W. Robert R. Zhang D. Teske K. A. Luo Y. Hanrahan J. W. (2007). Correctors of protein trafficking defects identified by a novel high-throughput screening assay. Chembiochem 8, 1012–1020 10.1002/cbic.200700027 17497613

[B11] Cheng S. H. Gregory R. J. Marshall J. Paul S. Souza D. W. White G. A. (1990). Defective intracellular transport and processing of CFTR is the molecular basis of most cystic fibrosis. Cell 63, 827–834 10.1016/0092-8674(90)90148-8 1699669

[B12] Dalemans W. Barbry P. Champigny G. Jallat S. Dott K. Dreyer D. (1991). Altered chloride ion channel kinetics associated with the delta F508 cystic fibrosis mutation. Nature 354, 526–528 10.1038/354526a0 1722027

[B13] Denning G. M. Anderson M. P. Amara J. F. Marshall J. Smith A. E. Welsh M. J. (1992). Processing of mutant cystic fibrosis transmembrane conductance regulator is temperature-sensitive. Nature 358, 761–764 10.1038/358761a0 1380673

[B14] Dormer R. L. Harris C. M. Clark Z. Pereira M. M. Doull I. J. Norez C. (2005). Sildenafil (Viagra) corrects DeltaF508-CFTR location in nasal epithelial cells from patients with cystic fibrosis. Thorax 60, 55–59 10.1136/thx.2003.019778 15618584 PMC1747155

[B15] Eisen M. B. Spellman P. T. Brown P. O. Botstein D. (1998). Cluster analysis and display of genome-wide expression patterns. Proc. Natl. Acad. Sci. U.S.A. 95, 14863–14868 10.1073/pnas.95.25.14863 9843981 PMC24541

[B16] French P. J. van Doorninck J. H. Peters R. H. Verbeek E. Ameen N. A. Marino C. R. (1996). A delta F508 mutation in mouse cystic fibrosis transmembrane conductance regulator results in a temperature-sensitive processing defect in vivo. J. Clin. Invest. 98, 1304–1312 10.1172/JCI118917 8823295 PMC507556

[B17] Gentleman R. C. Carey V. J. Bates D. M. Bolstad B. Dettling M. Dudoit S. (2004). Bioconductor: open software development for computational biology and bioinformatics. Genome Biol. 5, R80 10.1186/gb-2004-5-10-r80 15461798 PMC545600

[B18] Grynkiewicz G. Poenie M. Tsien R. Y. (1985). A new generation of Ca2+ indicators with greatly improved fluorescence properties. J. Biol. Chem. 260, 3440–3450 3838314

[B19] Hoyer K. Song Y. Wang D. Phan D. Balschi J. Ingwall J. S. (2011). Reducing the late sodium current improves cardiac function during sodium pump inhibition by ouabain. J. Pharmacol. Exp. Ther. 337, 513–523 10.1124/jpet.110.176776 21325441

[B20] Huang L. Li H. Xie Z. (1997). Ouabain-induced hypertrophy in cultured cardiac myocytes is accompanied by changes in expression of several late response genes. J. Mol. Cell. Cardiol. 29, 429–437 10.1006/jmcc.1996.0320 9140803

[B21] Huh J. R. Leung M. W. Huang P. Ryan D. A. Krout M. R. Malapaka R. R. (2011). Digoxin and its derivatives suppress T(H)17 cell differentiation by antagonizing RORgammat activity. Nature 472, 486–490 10.1038/nature09978 21441909 PMC3172133

[B22] Hutt D. M. Herman D. Rodrigues A. P. Noel S. Pilewski J. M. Matteson J. (2010). Reduced histone deacetylase 7 activity restores function to misfolded CFTR in cystic fibrosis. Nat. Chem. Biol. 6, 25–33 10.1038/nchembio.275 19966789 PMC2901172

[B23] Jensen T. J. Loo M. A. Pind S. Williams D. B. Goldberg A. L. Riordan J. R. (1995). Multiple proteolytic systems, including the proteasome, contribute to CFTR processing. Cell 83, 129–135 10.1016/0092-8674(95)90241-4 7553864

[B24] Johnson W. E. Li C. Rabinovic A. (2007). Adjusting batch effects in microarray expression data using empirical Bayes methods. Biostatistics 8, 118–127 10.1093/biostatistics/kxj037 16632515

[B25] Jurkuvenaite A. Chen L. Bartoszewski R. Goldstein R. Bebok Z. Matalon S. (2010). Functional stability of rescued delta F508 cystic fibrosis transmembrane conductance regulator in airway epithelial cells. Am. J. Respir. Cell Mol. Biol. 42, 363–372 10.1165/rcmb.2008-0434OC 19502384 PMC2830406

[B26] Kartner N. Hanrahan J. W. Jensen T. J. Naismith A. L. Sun S. Z. Ackerley C. A. (1991). Expression of the cystic fibrosis gene in non-epithelial invertebrate cells produces a regulated anion conductance. Cell 64, 681–691 10.1016/0092-8674(91)90498-N 1705179

[B27] Kunzelmann K. Schwiebert E. M. Zeitlin P. L. Kuo W. L. Stanton B. A. Gruenert D. C. (1993). An immortalized cystic fibrosis tracheal epithelial cell line homozygous for the delta F508 CFTR mutation. Am. J. Respir. Cell Mol. Biol. 8, 522–529 7683197 10.1165/ajrcmb/8.5.522

[B28] Lamb J. Crawford E. D. Peck D. Modell J. W. Blat I. C. Wrobel M. J. (2006). The Connectivity Map: using gene-expression signatures to connect small molecules, genes, and disease. Science 313, 1929–1935 10.1126/science.1132939 17008526

[B29] Li J. Zelenin S. Aperia A. Aizman O. (2006). Low doses of ouabain protect from serum deprivation-triggered apoptosis and stimulate kidney cell proliferation via activation of NF-kappaB. J. Am. Soc. Nephrol. 17, 1848–1857 10.1681/ASN.2006020130 16707566

[B30] Loo T. W. Bartlett M. C. Wang Y. Clarke D. M. (2006). The chemical chaperone CFcor-325 repairs folding defects in the transmembrane domains of CFTR-processing mutants. Biochem. J. 395, 537–542 10.1042/BJ20060013 16417523 PMC1462697

[B31] Lukacs G. L. Verkman A. S. (2012). CFTR: folding, misfolding and correcting the DeltaF508 conformational defect. Trends Mol. Med. 18, 81–91 10.1016/j.molmed.2011.10.003 22138491 PMC3643519

[B32] Ma T. Thiagarajah J. R. Yang H. Sonawane N. D. Folli C. Galietta L. J. (2002). Thiazolidinone CFTR inhibitor identified by high-throughput screening blocks cholera toxin-induced intestinal fluid secretion. J. Clin. Invest. 110, 1651–1658 10.1172/JCI16112 12464670 PMC151633

[B33] Matsumura Y. David L. L. Skach W. R. (2011). Role of Hsc70 binding cycle in CFTR folding and endoplasmic reticulum-associated degradation. Mol. Biol. Cell 22, 2797–2809 10.1091/mbc.E11-02-0137 21697503 PMC3154877

[B34] Newman R. A. Yang P. Pawlus A. D. Block K. I. (2008). Cardiac glycosides as novel cancer therapeutic agents. Mol. Interv. 8, 36–49 10.1124/mi.8.1.8 18332483

[B35] Nikolsky Y. Kirillov E. Zuev R. Rakhmatulin E. Nikolskaya T. (2009). Functional analysis of OMICs data and small molecule compounds in an integrated “knowledge-based” platform. Methods Mol. Biol. 563, 177–196 10.1007/978-1-60761-175-2_10 19597786

[B36] Nikolsky Y. Nikolskaya T. Bugrim A. (2005). Biological networks and analysis of experimental data in drug discovery. Drug Discov. Today 10, 653–662 10.1016/S1359-6446(05)03420-3 15894230

[B37] Norez C. Antigny F. Becq F. Vandebrouck C. (2006). Maintaining low Ca2+ level in the endoplasmic reticulum restores abnormal endogenous F508del-CFTR trafficking in airway epithelial cells. Traffic 7, 562–573 10.1111/j.1600-0854.2006.00409.x 16643279

[B38] Norez C. Antigny F. Noel S. Vandebrouck C. Becq F. (2009). A cystic fibrosis respiratory epithelial cell chronically treated by miglustat acquires a non-cystic fibrosis-like phenotype. Am. J. Respir. Cell Mol. Biol. 41, 217–225 10.1165/rcmb.2008-0285OC 19131642

[B39] Pedemonte N. Lukacs G. L. Du K. Caci E. Zegarra-Moran O. Galietta L. J. (2005). Small-molecule correctors of defective DeltaF508-CFTR cellular processing identified by high-throughput screening. J. Clin. Invest. 115, 2564–2571 10.1172/JCI24898 16127463 PMC1190372

[B40] Pilewski J. M. Frizzell R. A. (1999). Role of CFTR in airway disease. Physiol. Rev. 79, S215–S255 9922383 10.1152/physrev.1999.79.1.S215

[B41] Prassas I. Diamandis E. P. (2008). Novel therapeutic applications of cardiac glycosides. Nat. Rev. Drug Discov. 7, 926–935 10.1038/nrd2682 18948999

[B42] Rab A. Bartoszewski R. Jurkuvenaite A. Wakefield J. Collawn J. F. Bebok Z. (2007). Endoplasmic reticulum stress and the unfolded protein response regulate genomic cystic fibrosis transmembrane conductance regulator expression. Am. J. Physiol. Cell Physiol. 292, C756–C766 10.1152/ajpcell.00391.2006 16987996

[B43] Rennolds J. Boyaka P. N. Bellis S. L. Cormet-Boyaka E. (2008). Low temperature induces the delivery of mature and immature CFTR to the plasma membrane. Biochem. Biophys. Res. Commun. 366, 1025–1029 10.1016/j.bbrc.2007.12.065 18096515

[B44] Riordan J. R. Rommens J. M. Kerem B. Alon N. Rozmahel R. Grzelczak Z. (1989). Identification of the cystic fibrosis gene: cloning and characterization of complementary DNA. Science 245, 1066–1073 10.1126/science.2475911 2475911

[B45] Robert R. Carlile G. W. Liao J. Balghi H. Lesimple P. Liu N. (2010). Correction of the Delta phe508 cystic fibrosis transmembrane conductance regulator trafficking defect by the bioavailable compound glafenine. Mol. Pharmacol. 77, 922–930 10.1124/mol.109.062679 20200141

[B46] Robert R. Carlile G. W. Pavel C. Liu N. Anjos S. M. Liao J. (2008). Structural analog of sildenafil identified as a novel corrector of the F508del-CFTR trafficking defect. Mol. Pharmacol. 73, 478–489 10.1124/mol.107.040725 17975008

[B47] Rommens J. M. Iannuzzi M. C. Kerem B. Drumm M. L. Melmer G. Dean M. (1989). Identification of the cystic fibrosis gene: chromosome walking and jumping. Science 245, 1059–1065 10.1126/science.2772657 2772657

[B48] Routledge K. E. Gupta V. Balch W. E. (2010). Emergent properties of proteostasis-COPII coupled systems in human health and disease. Mol. Membr. Biol. 27, 385–397 10.3109/09687688.2010.524894 21054154

[B49] Sampson H. M. Robert R. Liao J. Matthes E. Carlile G. W. Hanrahan J. W. (2011). Identification of a NBD1-binding pharmacological chaperone that corrects the trafficking defect of F508del-CFTR. Chem. Biol. 18, 231–242 10.1016/j.chembiol.2010.11.016 21338920

[B50] Shmelkov E. Tang Z. Aifantis I. Statnikov A. (2011). Assessing quality and completeness of human transcriptional regulatory pathways on a genome-wide scale. Biol. Direct 6, 15 10.1186/1745-6150-6-15 21356087 PMC3055855

[B51] Sondo E. Tomati V. Caci E. Esposito A. I. Pfeffer U. Pedemonte N. (2011). Rescue of the mutant CFTR chloride channel by pharmacological correctors and low temperature analyzed by gene expression profiling. Am. J. Physiol. Cell Physiol. 301, C872–C885 10.1152/ajpcell.00507.2010 21753184 PMC3512166

[B52] Srivastava M. Eidelman O. Zhang J. Paweletz C. Caohuy H. Yang Q. (2004). Digitoxin mimics gene therapy with CFTR and suppresses hypersecretion of IL-8 from cystic fibrosis lung epithelial cells. Proc. Natl. Acad. Sci. U.S.A. 101, 7693–7698 10.1073/pnas.0402030101 15136726 PMC419668

[B53] Van Goor F. Hadida S. Grootenhuis P. D. Burton B. Stack J. H. Straley K. S. (2011). Correction of the F508del-CFTR protein processing defect in vitro by the investigational drug VX-809. Proc. Natl. Acad. Sci. U.S.A. 108, 18843–18848 10.1073/pnas.1105787108 21976485 PMC3219147

[B54] Van Goor F. Straley K. S. Cao D. Gonzalez J. Hadida S. Hazlewood A. (2006). Rescue of DeltaF508-CFTR trafficking and gating in human cystic fibrosis airway primary cultures by small molecules. Am. J. Physiol. Lung Cell. Mol. Physiol. 290, L1117–L1130 10.1152/ajplung.00169.2005 16443646

[B55] Varga K. Goldstein R. F. Jurkuvenaite A. Chen L. Matalon S. Sorscher E. J. (2008). Enhanced cell-surface stability of rescued DeltaF508 cystic fibrosis transmembrane conductance regulator (CFTR) by pharmacological chaperones. Biochem. J. 410, 555–564 10.1042/BJ20071420 18052931 PMC3939615

[B56] Wang J. Velotta J. B. McDonough A. A. Farley R. A. (2001). All human Na(+)-K(+)-ATPase alpha-subunit isoforms have a similar affinity for cardiac glycosides. Am. J. Physiol. Cell Physiol. 281, C1336–C1343 11546672 10.1152/ajpcell.2001.281.4.C1336

[B57] Wang X. Koulov A. V. Kellner W. A. Riordan J. R. Balch W. E. (2008). Chemical and biological folding contribute to temperature-sensitive DeltaF508 CFTR trafficking. Traffic 9, 1878–1893 10.1111/j.1600-0854.2008.00712.x 18764821 PMC2683680

[B58] Wu X. Wakefield J. K. Liu H. Xiao H. Kralovics R. Prchal J. T. (2000). Development of a novel trans-lentiviral vector that affords predictable safety. Mol. Ther. 2, 47–55 10.1006/mthe.2000.0126 10899827

[B59] Yang P. Menter D. G. Cartwright C. Chan D. Dixon S. Suraokar M. (2009). Oleandrin-mediated inhibition of human tumor cell proliferation: importance of Na,K-ATPase alpha subunits as drug targets. Mol. Cancer Ther. 8, 2319–2328 10.1158/1535-7163.MCT-08-0749 19671733

[B60] Yoo J. S. Moyer B. D. Bannykh S. Yoo H. M. Riordan J. R. Balch W. E. (2002). Non-conventional trafficking of the cystic fibrosis transmembrane conductance regulator through the early secretory pathway. J. Biol. Chem. 277, 11401–11409 10.1074/jbc.M200382200 11799116

[B61] Zhang L. Button B. Gabriel S. E. Burkett S. Yan Y. Skiadopoulos M. H. (2009). CFTR delivery to 25% of surface epithelial cells restores normal rates of mucus transport to human cystic fibrosis airway epithelium. PLoS Biol. 7, e1000155 10.1101/cshperspect.a009589 19621064 PMC2705187

[B62] Zhang S. Malmersjo S. Li J. Ando H. Aizman O. Uhlen P. (2006). Distinct role of the N-terminal tail of the Na,K-ATPase catalytic subunit as a signal transducer. J. Biol. Chem. 281, 21954–21962 10.1074/jbc.M601384200 16723354

